# Systematic scoping review of external validation studies of AI pathology models for lung cancer diagnosis

**DOI:** 10.1038/s41698-025-00940-7

**Published:** 2025-06-07

**Authors:** Soumya Arun, Mariia Grosheva, Mark Kosenko, Jan Lukas Robertus, Oleg Blyuss, Rhian Gabe, Daniel Munblit, Judith Offman

**Affiliations:** 1 Centre for Cancer Screening, Prevention and Early Diagnosis, Wolfson Institute of Population Health, Queen Mary University of London, London, UK; 2 Department of Paediatrics and Paediatric Infectious Diseases, Institute of Child’s Health, I.M. Sechenov First Moscow State Medical University, Sechenov University, Moscow, Russia; 3 Department of Histopathology, Royal Brompton and Harefield, Guy’s and St Thomas’ NHS Foundation Trust, London, UK; 4 Faculty of Medicine, National Heart and Lung Institute, Imperial College London, London, UK; 5 Centre for Evaluation and Methods, Wolfson Institute of Population Health, Queen Mary University of London, London, UK; 6 Division of Care in Long Term Conditions, Florence Nightingale Faculty of Nursing, Midwifery and Palliative Care, King’s College London, London, UK

**Keywords:** Epidemiology, Diagnosis, Cancer imaging, Cancer screening, Lung cancer, Computational biology and bioinformatics

## Abstract

Clinical adoption of digital pathology-based artificial intelligence models for diagnosing lung cancer has been limited, partly due to lack of robust external validation. This review provides an overview of such tools, their performance and external validation. We systematically searched for external validation studies in medical, engineering and grey literature databases from 1st January 2010 to 31st October 2024. 22 studies were included. Models performed various tasks, including classification of malignant versus non-malignant tissue, tumour growth pattern classification and subtyping of adeno- versus squamous cell carcinomas. Subtyping models were most common and performed highly, with average AUC values ranging from 0.746 to 0.999. Although most studies used restricted datasets, methodological issues relevant to the applicability of models in real-world settings included small and/or non-representative datasets, retrospective studies and case-control studies without further real-world validation. Ultimately, more rigorous external validation of models is warranted for increased clinical adoption.

## Introduction

Digital pathology refers to the analysis, management and sharing of pathology-related data within a digital environment^1^. The advent of digital pathology has driven the development of numerous artificial intelligence (AI) models for application on digital pathology images to aid cancer diagnosis. Such AI tools are being developed at an increasing rate every year. Lung cancer is the leading cause of cancer-related death in the UK, accounting for approximately 35, 000 deaths annually^2^. This high mortality rate is largely a result of late-stage diagnosis. Notably, the five-year survival rate for individuals diagnosed with lung cancer at stage 1 is 65%, however this decreases considerably to 5% for individuals diagnosed at stage 4^3^. The implementation of national targeted lung cancer screening programmes in the UK and other high-income countries worldwide may improve patient outcomes^4^. Nevertheless, increased screening is likely to result in increased referrals to pathology services, and place substantial strain on an already-burdened workforce^5^. AI could potentially address these workforce bottlenecks^6^.

The application of AI models to digitised whole slide images (WSIs) is revolutionising cancer diagnosis. Pathologists are facing considerable pressures with rising workloads and a need to analyse increasingly complex and vast datasets. By automating certain tasks, AI models could complement pathologists in their clinical workflows and offer scalable diagnostic support^4^. Importantly, AI is capable of rapidly analysing vast datasets and may recognise patterns that may not be easily discernible to the human eye^7^. This ability is especially pertinent in lung cancer, where early diagnosis can lead to a substantial improvement in patient outcomes^8^. An emerging trend in the field of AI is the development of foundation models. These are large-scale models that are trained on vast datasets and act as a foundation for a diverse range of downstream tasks^9^. Notably, several histopathology-based AI models have already been approved by the FDA, including Paige Prostate for facilitating the diagnosis of prostate cancer^10^.

Despite their potential, the clinical adoption of cancer diagnostic AI pathology tools has been extremely limited to date. This is largely attributable to lack of robust external validation of models prior to deployment, and concerns regarding the generalisability of models to real-world clinical settings^11^. External validation refers to the evaluation of model performance using data taken from a separate source to the data used for training and testing the model^11^. A major challenge to widespread clinical adoption of these models is the problem of validating these tools using diverse, real-world datasets^11^. While AI models may perform well on internal datasets, their performance may drop considerably on external datasets that reflect the variability encountered in clinical practice. Robust external validation is important for assessing the generalisability of a model to different patient populations and is a critical step before AI models can be trusted and integrated into clinical workflows^12^.

Current literature on external validation is sparse and existing systematic reviews evaluating validation studies for pathology-based lung cancer diagnostic algorithms focus primarily on AI techniques and validation on internal datasets^13–15^. A review of external validation studies for these AI tools, with a focus on methodological robustness, is yet to be conducted. Our review provides an overview of models used to facilitate lung cancer diagnosis from digital pathology images and explores the current state of external validation of these models. The primary objective of this review was to assess the methodological robustness of validation studies and report model performance where possible, focusing exclusively on external, independent datasets. We chose to use a systematic scoping review approach to map the available evidence, identify gaps in the evidence, and critically appraise included studies.

## Results

**Fig. 1:**
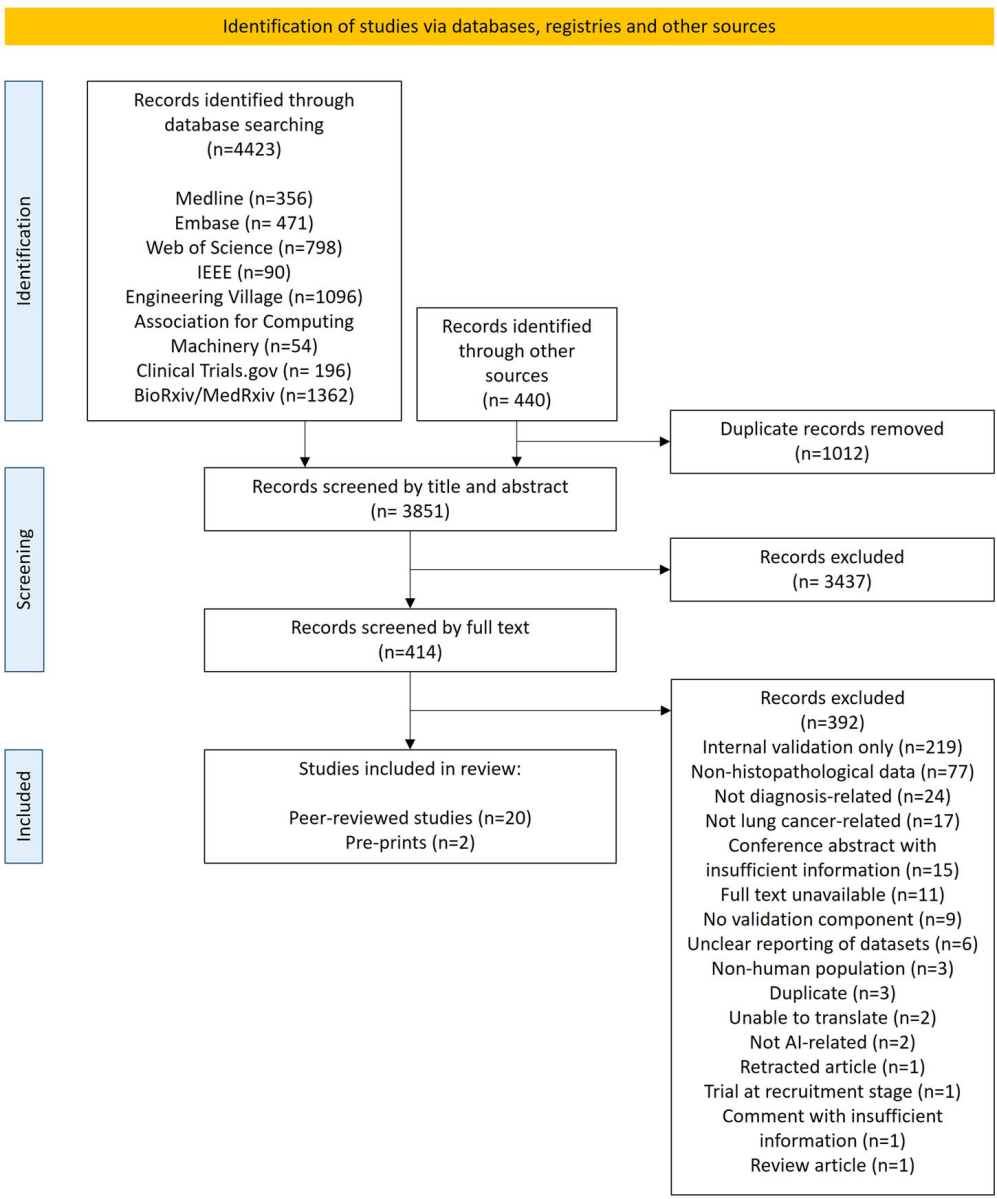
PRISMA flow diagram. Summary of studies included and excluded at each stage of the review.

Database searches resulted in 4423 studies, with 440 additional studies identified through other sources, including Google Scholar and a snowballing approach (Fig.1). After duplicates were removed, we screened 3851 titles and abstracts, and reviewed 414 full-text articles. Overall, 22 studies met the inclusion criteria, including 20 publications and two preprints (Table 1). During the screening process, we identified 239 papers describing the development and validation of pathology lung cancer detection models. It is noteworthy that approximately only 10% of these papers described the external validation of models.

**Table 1:** Publications of studies that met the inclusion criteria, task(s) performed by each model, external validation dataset details and algorithm details for each study

Publication Author and Date	Publication Title	Model Task	External Validation Dataset Details			Algorithm Details	
			Number of samples	Public dataset, or restricted dataset and name of public dataset(s) if used	Number of centres that restricted datasets were taken from and country	Algorithm name	Code availability
Bilaloglu et al. 2019^a^^17^	Efficient pan-cancer whole-slide image classification and outlier detection using convolutional neural networks	•Subtyping •Classification of malignant versus non-malignant tissue	340	Restricted	One academic medical centre in the US	PathCNN	https://github.com/sedab/PathCNN
Borras Ferris et al. 2024^32^	A full pipeline to analyse lung histopathology images	•Subtyping •Classification of malignant versus non-malignant tissue	1036	Public TCGA			Unavailable
Cao et al. 2023^18^	E2EFP-MIL: End-to-end and high-generalizability weakly supervised deep convolutional network for lung cancer classification from whole slide image	•Subtyping	1583	Restricted	Three hospitals in China	E2EFP-MIL	https://github.com/raycaohmu/E2EFP-MIL
Chen et al. 2022^19^	A whole-slide image (WSI)-based immunohistochemical feature prediction system improves the subtyping of lung cancer	•Subtyping •Classification of malignant versus non-malignant tissue •Biomarker identification	299	Restricted	One academic medical centre in China	WIFPS	https://ngdc.cncb.ac.cn/biocode/tools/BT007223
Coudray et al. 2018^20^	Classification and mutation prediction from non–small cell lung cancer histopathology images using deep learning	•Subtyping •Classification of malignant versus non-malignant tissue	340	Restricted	One academic medical centre in the US		https://github.com/ncoudray/DeepPATH
Gertych et al. 2019^33^	Convolutional neural networks can accurately distinguish four histologic growth pattens of lung adenocarcinoma in digital slides	•Classification of malignant versus non-malignant tissue •Classification of tumour growth pattern	27	Public TCGA			https://github.com/zhaoxuanma/Deeplearning-digital-pathology
Hari et al. 2021^a^^21^	Examining batch effect in histopathology as a distributionally robust optimisation problem	•Subtyping •Classification of malignant versus non-malignant tissue	Unclear	Public TCGA	Four academic medical centres, one cancer centre and one hospital in the US	ERM model	Unavailable
Kanavati et al. 2020^34^	Weakly-supervised learning for lung carcinoma classification using deep learning	•Classification of malignant versus non-malignant tissue	1670 Public: 1170 Restricted: 500	Mixed TCGA, TCIA	One hospital in Japan		Unavailable
Kanavati et al. 2021^22^	A deep learning model for the classification of indeterminate lung carcinoma in biopsy whole slide images	•Subtyping •Classification of malignant versus non-malignant tissue	1405 Public: 905 Restricted: 500	Mixed TCGA	One hospital in Japan		Unavailable
Le Page et al. 2021^23^	Using a convolutional neural network for classification of squamous and non-squamous non-small cell lung cancer based on diagnostic histopathology HES images	•Subtyping	65	Restricted	One academic medical centre in France		Available from author upon reasonable request
Lu et al. 2021^24^	Data Efficient and Weakly Supervised Computational Pathology on Whole Slide Images	•Subtyping	241	Restricted	One hospital in the US	CLAM	https://github.com/mahmoodlab/CLAM
Mukashyaka et al. 2024^25^	SAMPLER: unsupervised representations for rapid analysis of whole slide tissue images	•Subtyping	Unclear	Public TCGA, CPTAC		SAMPLER	https://figshare.com/articles/software/SAMPLER_basic_code_example/23713404
Noorbakhsh et al. 2020^26^	Deep learning-based cross-classifications reveal conserved spatial behaviours within tumour histological images	•Subtyping •Classification of malignant versus non-malignant tissue	2115	Public CPTAC			https://github.com/javadnoorb/HistCNN
Quiros et al. 2024^27^	Mapping the landscape of histomorphological cancer phenotypes using self-supervised learning on unannotated pathology slides	•Subtyping	138	Restricted	One academic medical centre in the US	PRL	https://github.com/AdalbertoCq/Histomorphological-Phenotype-Learning
Sakamoto et al. 2022^35^	A collaborative workflow between pathologists and deep learning for the evaluation of tumour cellularity in lung adenocarcinoma	•Classification of malignant versus non-malignant tissue •Prediction of tumour cellularity	125	Restricted	Two hospitals in Japan		Available from author upon reasonable request
Sharma et al. 2024^31^	Optimizing Knowledge Transfer in Sequential Models: Leveraging Residual Connections in Flow Transfer Learning for Lung Cancer Classification	•Classification of malignant versus non-malignant tissue •Subtyping	566	Public TCGA			Unavailable
Swiderska-Chadaj et al. 2020^36^	A deep learning approach to assess the predominant tumour growth pattern in whole-slide images of lung adenocarcinoma	•Classification of malignant versus non-malignant tissue •Classification of tumour growth pattern	20	Restricted	One academic medical centre in the Netherlands	DenseNet	Unavailable
Vorontsov et al. 2024^16^	A foundation model for clinical-grade computational pathology and rare cancers detection	•Classification of malignant versus non-malignant tissue •Biomarker identification	Unclear	Restricted	Unknown	Virchow	https://huggingface.co/paige-ai/Virchow
Wang et al. 2019^37^	ConvPath: A software tool for lung adenocarcinoma digital pathological image analysis aided by a convolutional neural network	•Classification of cell types	130	Restricted	Unknown	ConvPath	https://qbrc.swmed.edu/projects/cnn/
Wang et al. 2023^28^	Deep Learning of Cell Spatial Organizations Identifies Clinically Relevant Insights in Tissue Images	•Subtyping	496	Restricted	Unknown	Ceograph	https://github.com/sdw95927/Ceograph/
Yang et al. 2021^29^	Deep learning-based six-type classifier for lung cancer and mimics from histopathological whole slide images: a retrospective study	•Subtyping •Classification of malignant versus non-malignant tissue	634 Public: 422 Restricted: 212	Mixed TCGA	One hospital in China		Available from author upon reasonable request
Yu et al. 2020^30^	Classifying non-small cell lung cancer histopathology types and transcriptomic subtypes using convolutional neural networks	•Subtyping •Classification of malignant versus non-malignant tissue	125	Public ICGC			https://github.com/khyu/lung-CNN

*TCGA* The Cancer Genome Atlas, *TCIA* The Cancer Imaging Archive, *CPTAC* Clinical Proteomic Tumor Analysis Consortium, *ICGC* International Cancer Genome Consortium.

^**a**^Preprint article.

### AI models and tasks

**Fig. 2:**
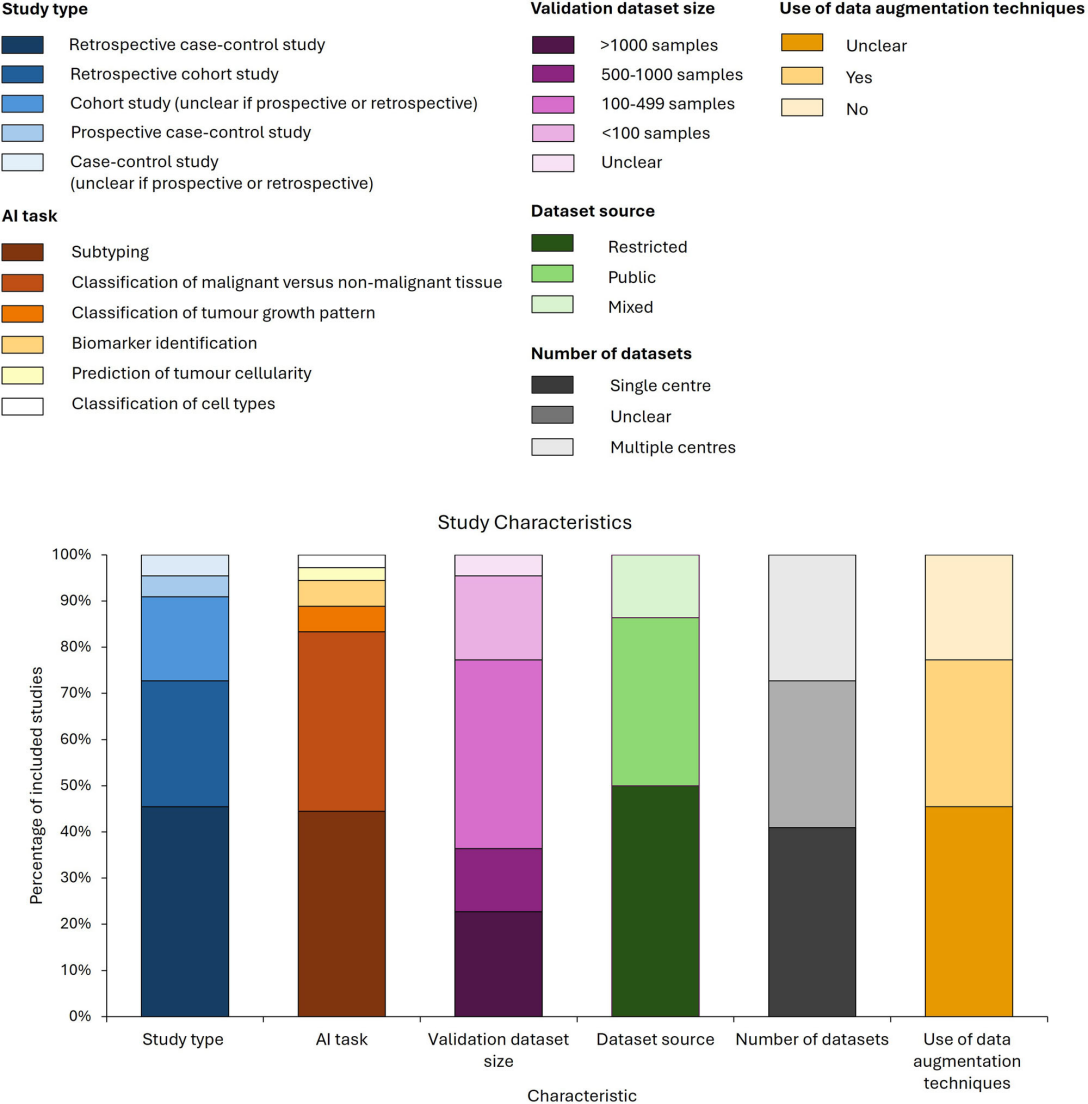
Characteristics of included studies.

Figure 2 presents the characteristics of the included studies. 18 models facilitated the diagnosis of non-small cell lung cancer (NSCLC), focusing primarily on lung adenocarcinoma (LUAD) and/or lung squamous cell carcinoma (LUSC). Three models detected small cell lung cancer (SCLC) in addition to NSCLC. We identified one foundation model, named Virchow, which was trained for pan-cancer detection using approximately 1.5 million WSIs covering 17 tissue types^16^. Virchow was trained and validated on lung cancer tissue, however the lung cancer subtypes intended to be detected by the model were not specified^16^.

Models performed various tasks along the diagnostic pathway, most commonly subtyping (n = 16)^17–32^. 13 subtyping models distinguished LUAD from LUSC, whereas three models distinguished LUAD, LUSC and SCLC. Other tasks performed by AI models included classification of malignant versus non-malignant tissue (n = 14)^17,19–22,26,29–36^, tumour growth pattern classification (n = 2)^33,36^, biomarker identification (n = 2)^16,19^, prediction of tumour cellularity (n = 1)^35^, and classification of cell types (n = 1)^37^. We identified 14 multi-tasking models^16,17,19–22,26,29–36^, the majority of which (n = 10) combined subtyping and classification of malignant versus non-malignant tissue^17,19–22,26,29–32^.

Information regarding the intended role of the model within the diagnostic pathway, the intended clinical setting and the intended country of deployment was limited. Three authors provided details on the intended clinical setting of their models, such as country of deployment and whether the target population would be asymptomatic or symptomatic^18,30,35^. One author reported that their model could act as a triage tool in clinical practice^16^, whereas authors of 12 studies reported that their AI model was developed to aid the clinician without providing any further details^17–23,30,32,33,35–37^. One author reported that their study was for research purposes only and that the model was not developed specifically as a clinical tool^26^.

### Study type

16 out of 22 studies were retrospective^16,20–26,28–34,37^, with retrospective case-control studies being the most used study design (n = 10)^16,20–22,26,29–31,33,34^. We identified one prospective case-control study^36^, however we could not identify any completed prospective cohort studies or randomised controlled trials. For five studies, it was unclear whether data was collected retrospectively or prospectively^17–19,27,35^.

### Datasets

Histopathological datasets used for external validation were heterogeneous in size, with studies using as few as 20 samples to as many as 2115 samples (see Table 1). Around half of the studies (n = 9) used datasets consisting of between 100 and 500 images^17,19,20,24,27,28,30,35,37^. Similarly to dataset size, the number of datasets used, and the source of datasets varied considerably. Seven studies were single-centre studies^17,19,20,23,24,27,37^, whereas six studies used images from multiple centres, ranging from two centres to four centres^18,21,22,29,34,35^. While most studies used images from restricted datasets from secondary care hospitals and tertiary centres^16–20,23,24,27,28,35–37^, three studies used a combination of both public and restricted datasets^22,29,34^.

### Technical diversity within datasets

Over half of the studies (n = 12) used techniques to address potential variations in images that may arise due to differences in equipment or tissue processing protocols across centres. Nine studies reported using either one or a combination of the following to increase technical diversity in datasets: WSIs created with different whole slide scanners, various magnifications, slides preserved using different methods (e.g. FFPE or frozen), different tissue samples (e.g. biopsies or resections), slides prepared with various stains, and slides containing artefacts (e.g. bubbles and scratches)^17,18,20,22,24,25,33–35^. Among the 13 studies where the use of these methods was unclear, two studies simulated technical diversity through data augmentation techniques such as rotation, flipping, and varying brightness, saturation, contrast, and hue^21,37^. Conversely, three studies used stain normalisation to minimise variability between images^18,23,27^.

### Quality assessment

**Fig. 3:**
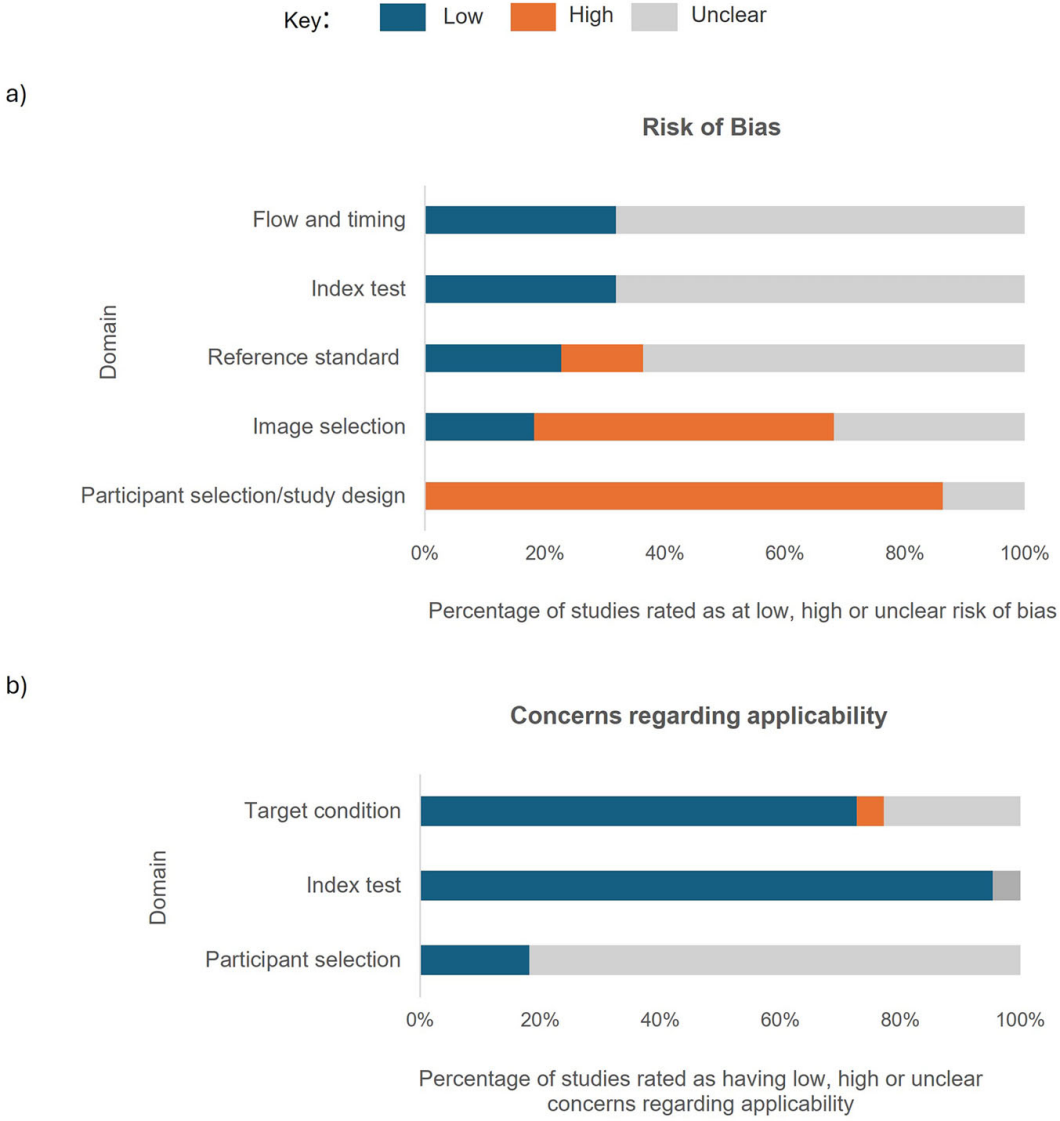
Results of the quality assessment conducted using QUADAS-AI-P. **a** Results of the risk of bias assessment. For each QUADAS-AI-P domain, the blue, orange and grey sections of the bar indicate the percentage of studies judged to be at low, high or unclear risk of bias, respectively. **b** Results of the concerns regarding applicability assessment. For each QUADAS-AI-P domain, the blue, orange and grey sections of the bar indicate the percentage of studies considered to have low, high or unclear concerns regarding applicability. QUADAS-AI-P: QUality Assessment tool of Diagnostic Accuracy Studies tailored to Artificial Intelligence and digital Pathology.

High or unclear risk of bias was observed for all studies in at least one of the five assessed QUADAS-AI-P domains. As depicted in Fig. 3, high risk of bias was noted for 14% of studies in the ‘Reference standard’ domain, 50% of studies in the ‘Image selection’ domain, and 86% of studies in the ‘Participant selection/study design’ domain. On the other hand, low risk of bias was noted for 18% of studies in the ‘Image selection’ domain, 23% of studies in the ’Reference standard’ domain, and 32% of studies in both the ‘Flow and timing’ and ‘Index test’ domains. Due to inadequate reporting, the risk of bias was unclear for most studies in the ‘Flow and timing’, ‘Index test’, and ‘Reference standard’ domains. Additionally, concerns regarding applicability were high for one study in the ‘Target condition’ domain, low for 95% of studies in the ‘Index test’ domain and unclear for 82% of studies in the ‘Participant selection’ domain due to insufficient reporting of participant characteristics (Fig. 3). The risk of bias ratings and concerns regarding applicability are shown for each individual study in Supplementary material 3. See supplementary material 4 for a full list of methodological concerns.

### Diagnostic performance and evaluation metrics used

The area under the receiver operating characteristic curve (AUC) was reported in 17 out of 22 studies^17–20,22–32,34^, making it the most commonly reported evaluation metric overall. Notably, only four studies reported sensitivity and/or specificity^16,19,20,29^. Other metrics used to evaluate models included accuracy, F1 score, precision, recall, and area under the precision-recall curve (AUPRC). Performance metrics were reported according to dataset, tissue type and/or preservation method, lung cancer subtype or unit of analysis (patch-level or slide-level). Importantly, we could not conduct a meta-analysis due to considerable heterogeneity in AI task, evaluation metrics used, unit of analysis and reporting. It was only possible to compare performance metrics for models that subtyped lung cancers, as this was the most common, most clearly defined task with the greatest consistency in reporting (Table 2). Models for subtyping lung cancers performed highly, with average AUC values ranging from 0.746 (Mukashyaka et al. 2024) to 0.999 (Kanavati et al. 2021)^22,25^. Notably, out of the 16 studies evaluating models for subtyping lung cancers, eight provided ROC curves^20,22–24,27–30^, and eight provided measures of variability^20–22,25,27,29,30,32^.

**Table 2:** Performance of lung cancer subtyping models

Study	Lung cancer subtype	Evaluation Metric			
		AUC	Sensitivity	Specificity	Other
Bilaloglu et al. (2019) ^a^^17^	LUAD	0.88^b,c^	–	–	–
	LUSC	0.93^b,c^			
Borras Ferris et al. (2024)^32^	LUAD vs LUSC	0.9433 ± 0.0198	–	–	F1 score: 0.7726 ± 0.0438
Cao et al. (2023)^18^	LUAD vs LUSC	0.945^d^	–	–	AUPRC: 0.946^d^
Chen et al. (2022)^19^	LUAD vs LUSC vs SCLC	–	0.831	0.912	Accuracy: 0.858
Coudray et al. (2018)^20^	LUAD	0.874 (95% CI, 0.803–0.930)^b,c^	–	–	–
	LUSC	0.932 (95% CI, 0.883–0.971)^b,c^			
Hari et al. (2021) ^a^^21^	LUAD vs LUSC	–	–	–	F1 score: 0.81 ± 0.12
Noorbakhsh et al. (2020)^26^	LUAD	0.97^c^	–	–	–
	LUSC	0.96^c^			
Kanavati et al. (2021)^22^	LUAD	0.952 (95% CI, 0.933–0.967)^d^	–	–	–
	LUSC	0.968 (95% CI, 0.952-0.982)^d^			
	SCLC	0.999 (95% CI, 0.993–1.0)			
Le Page et al. (2021)^23^	LUAD vs LUSC	0.81	–	–	Accuracy: 0.85
Lu et al. (2021)^24^	LUAD vs LUSC	0.917^c^	–	–	–
Mukashyaka et al. (2024)^25^	LUAD vs LUSC	0.746 ± 0.018	–	–	–
Quiros et al. (2024)^27^	LUAD vs LUSC	0.98 (95% CI, 0.97–0.99)	–	–	–
Sharma et al. (2024)^31^	LUAD vs LUSC	0.996	–	–	Accuracy: 97%
Wang et al. (2023)^28^	LUAD vs LUSC	0.90	–	–	Accuracy: 99%
Yang et al. (2021)^29^	LUAD vs LUSC vs SCLC	0.973 (95% CI, 0.964–0.980)^d^	0.823^d^	–	F1 score: 0.833^d^ Precision: 0.873^d^
Yu et al. (2020)^30^	LUAD vs LUSC	0.864 ± 0.027	–	–	–

^a^Preprint article.

^b^Weighted average based on number of samples of a certain tissue type.

^c^Weighted average based on number of samples of a certain lung cancer subtype.

^d^Weighted average based on validation dataset sizes.

## Discussion

To our knowledge, this is the most up-to-date systematic scoping review evaluating the methodological robustness of studies externally validating AI models for diagnosing lung cancer from digital pathology images. We identified 20 publications and two preprints. Models performed various tasks to facilitate lung cancer diagnosis, with subtyping being the most common task. Models for subtyping lung cancers performed well, with average AUC values ranging from 0.746 (Mukashyaka et al. 2024) to 0.999 (Kanavati et al. 2021)^22,25^.

Promisingly, over 60% of studies used at least one restricted external validation dataset. Restricted datasets are advantageous over public datasets as it is easier to assess the reliability of ground truth labels and to ensure that validation is truly external^38^. Images sometimes overlap between online repositories, so even if models were trained and validated on separate public datasets, these datasets may not be completely independent^38^.

Nevertheless, we identified several methodological issues regarding the external validation process. These include failure to account for technical variation across centres and poor reporting of clinically meaningful evaluation metrics. Furthermore, only one prospective real-world validation study has been conducted to date. Other studies mostly used retrospective, non-representative datasets and case-control study designs, reflecting early-stage validation.

Notably, over 80% of studies were at high risk of bias in the ‘Participant selection/study design’ domain, primarily due to the use of non-diverse, retrospective datasets and case-control studies which are highly susceptible to spectrum bias^39^. Separate recruitment of cases and controls may result in those with less extreme phenotypes (e.g. early-stage, asymptomatic individuals) being missed from datasets. Subsequently, algorithms may perform inadequately on these individuals in clinic. Spectrum bias is a particular concern for algorithms designed to be used in screening settings, where a larger proportion of early-stage cancers will be identified compared to a symptomatic population. This is of particular importance with the introduction of lung cancer screening programmes in high-income countries worldwide^4^.

While retrospective validation is time- and cost-effective, models may underperform in real-world settings. Prospective studies and ongoing monitoring would be beneficial for understanding whether a model works with existing infrastructure and scanners, and on a population reflecting the target population as closely as possible. Prospective studies could range from small-scale implementation studies to larger RCTs. Encouragingly, we identified a clinical trial at the recruitment stage aiming to validate a lung cancer detection model using a prospective cohort study design (NCT05925764).

Another methodological concern was lack of diversity within the study population. Among the three studies that reported participant ethnicity, only a minority of participants were non-White. Research indicates racial disparities in lung cancer subtype and stage^40^. For example, Black individuals have a higher NSCLC incidence rate and are more likely to present with advanced lung cancer compared to White individuals^40,41^. Notably, none of the studies performed sub-group analysis by ethnicity.

Importantly, model performance is affected by sample size^42^. It is notable that 12 studies used fewer than 500 samples^17,19,20,23,24,27,30,33,35–37,43^, and that sample size was not reported for three studies^16,21,25^. Although it is encouraging that five studies used over 1000 samples^17,21,25,31,34^, dataset size may not necessarily reflect diversity within a dataset. The number of participants in these studies were not reported, and multiple samples may have originated from the same participant. Furthermore, around 90% of studies failed to report the proportion of subtypes and stages represented in datasets. Although two authors used data augmentation techniques to increase image variability, the level of technical diversity within datasets was concerning for half of the included studies^16,19,23,26–32,36^. Out of six single-centre studies, two studies applied stain normalisation to standardise stain colour^23,27^, and one study failed to report the use of any methods to increase technical variation^19^. This poses the risk of suboptimal performance on data from external centres using different equipment and/or tissue processing protocols. See recommendations in Box 1 below for methods to increase technical diversity within histopathological datasets.

In contrast with previous studies, clinically meaningful evaluation metrics such as sensitivity and specificity were poorly reported. Many authors failed to consider the clinical application of their models, and therefore did not determine suitable levels of sensitivity or specificity required to select optimal cutoffs from ROC curves. Moreover, sensitivity and specificity values were difficult to calculate as confusion matrices and ROC curves were rarely provided. With the increasing development of pathology AI models for cancer detection, more standardised reporting of metrics would enable future meta-analyses to determine the most effective model for a particular task.

Our findings that lung cancer subtyping was the most common task, and that AUC was the most frequently reported metric, echo the results of Prabhu et al. (2022) and Davri et al. (2023), respectively^13,14^. Importantly, previous reviews evaluating both internal and external validation studies together highlighted issues with small datasets, lack of multicentre studies and heterogeneity in metrics used^13–15^. Our results indicate that these issues persist even when considering external validation studies alone. With regards to the quality assessment, similarly to McGenity et al.^15^, we found that the greatest number of studies were at high or unclear risk of bias in the ‘Participant selection/study design’ domain. While McGenity et al.^15^ attributed this primarily to lack of non-random, non-consecutive and unclear participant enrolment, we found that this was predominantly due to lack of diversity within the participant population. In contrast with McGenity et al.^15^, the quality assessment tool we used contained an additional domain titled ‘Image selection’. We identified eleven studies at high risk of bias in this domain due to lack of technical diversity within datasets^17,19–21,23,25–27,30,31,33^.

Our review has several strengths. Firstly, given the fast-paced nature of the field, this is the most comprehensive and up-to-date review of its kind. While previous research has been limited to models for LUAD and LUSC^14^, we additionally included models that facilitated the diagnosis of any lung cancer subtype. Moreover, we used a broad, comprehensive search strategy and explored both engineering and medical databases. Secondly, we adhered to PRISMA-ScR guidance and published our protocol a priori, outlining any changes in an updated version^44^. Thirdly, screening, data extraction and the quality assessment were independently conducted by at least two reviewers, and conflicts at the screening stage were resolved independently by a third reviewer. Nevertheless, a key limitation of this review was the inability to conduct a meta-analysis due to heterogeneity in evaluation metrics used across studies. Additionally, our quality assessment was hindered by poor reporting and lack of response to missing information requests. Out of the 22 authors contacted for missing information, only seven authors responded. Finally, it is noteworthy that preprints were included in the review and that relevant literature may have been missed as we were unable to translate two studies.

Lung cancer subtyping models may bring considerable benefits to clinical practice. Firstly, by automating certain tasks using AI, stains and other materials can be conserved for downstream tasks such as genomic analysis and treatment planning. Secondly, LUAD and LUSC require different treatment modalities^45^. While, targeted therapies such as EGFR inhibitors and BRAF inhibitors are effective for treating LUAD, these therapies have shown limited benefit for LUSC^45,46^. Treatment for LUSC typically involves surgery, radiotherapy and platinum-based chemotherapy, which are most effective for early-stage disease^45^. Hence, timely and accurate lung cancer subtyping is critical. A delay in treatment could result in rapid disease progression, making treatment more complex and increase the risk of side effects^47^.

In conclusion, this review provides an overview of the landscape of AI models for the digital pathology-based diagnosis of lung cancer and their external validation. Such tools have great potential to support clinical workflows and may be best positioned as triage tools or as add-on tools to augment pathologists. The field is evolving rapidly, however, robust clinical validation to date is lacking, which raises concerns about whether model performance would be maintained in real-world clinical settings. The methodological issues identified in this review highlight the need for more rigorous external validation of pathology lung cancer detection models for increased clinical adoption. Based on these issues, we propose a set of recommendations for more robust external validation to aid the translation of AI cancer detection models that are safe, ethical and effective in clinical practice (Box 1). Further work is warranted to clarify exact requirements for a robust external validation dataset. For example, expert consensus may help to determine the required sample size, number of contributing centres, and the optimal level of geographic and demographic diversity. This review focuses on lung cancer as a use case; however, our findings are likely to apply to other cancer types as well.

Box 1 Proposed recommendations for the external validation of pathology AI models to facilitate cancer diagnosis (based on methodological concerns in Supplementary Material 4) **Algorithm**

*Design recommendations:*

Define:

- Intended clinical setting (e.g. country, population demographics, cancer stage and cancer subtype).
- Role of the model within the diagnostic pathway (e.g. aid for the clinician, replacement or triage tool).

**Study design**

*Design recommendations:*

- Data should be taken from a separate source to the data used to train the model.
- Case-control studies should be followed with prospective cohort or implementation studies and/or RCTs to avoid potential spectrum bias.
- Retrospective studies should use restricted datasets if possible.
- Data should be taken from multiple centres and geographic locations, if possible, as these are likely to differ in their population, protocols and technical equipment.
  ○ As a minimum, models should be validated in their intended clinical setting prior to deployment. Further local validation will allow assessment of whether a model’s performance translates to a specific centre.
- The minimum sample size needed for a maximum acceptable sampling error should be calculated a priori. Several methods for sample size determination have been proposed^51,52^.
  ○ The sample size should be determined with regard to the clinical outcome to be measured.
  ○ The dataset should be large enough to include variations in disease encountered in clinical practice, including rare pathologies.
  ○ If possible, the dataset should be powered for subgroup analysis (e.g. by age, sex, ethnicity, cancer stage, cancer subtype).
  ○ Dataset size may be artificially increased through data augmentation techniques. Such methods may be of particular value for rare cancers with small patient cohorts.

- Use an appropriate ground truth. Several pathologists with a high level of expertise would be considered the gold-standard.

*Reporting recommendations:*

- Study design (prospective or retrospective, case-control, cohort study or RCT).
- Number of samples used for external validation.
- Number of centres that data was taken from.
- Ground truth, including whether set by another algorithm.

**Population/participant selection**

*Design recommendations:*

- The distribution of subgroups (e.g. age, sex, cancer stage and cancer subtype) within a dataset should ideally reflect the distribution of subgroups in the target population.
  ○ For example, in the UK, approximately half of lung cancer cases occur in males and half occur in females^53^. These proportions should be reflected in the external validation dataset.

*Reporting recommendations:*

- Target population (e.g. symptomatic or asymptomatic individuals).
- Number of participants included in the final analysis.
- Demographic characteristics of the population (e.g. age, sex, ethnicity, sociodemographic status).
- Sample collection method (e.g. random, consecutive).
- Number of samples of each cancer stage and subtype.
- If certain sub-groups were missed out from external validation datasets (e.g. certain stages, subtypes, ethnicities), include reasons for their missingness.

**Image selection**

*Design recommendations:*

- The dataset should represent technical variations encountered in real-world settings. Technical diversity could be introduced through the use of:
  ○ samples prepared with different protocols (e.g. staining techniques, preservation methods)
  ○ samples scanned with different whole slide scanners.
  ○ samples with artefacts (e.g. bubbles, folds, pen markings)
  ○ samples scanned at different magnifications
  ○ different tissue samples (e.g. biopsies, resections)
  ○ data augmentation techniques
  ○ generative adversarial networks

- Contemporary datasets should be used, if possible, as these are most likely to reflect current tissue processing protocols and scanning procedures.
- Where there is limited access to contemporary data, historical data may be used. In such cases, the potential for dataset drift and bias should be considered.

*Reporting recommendations:*

- Number of samples taken from each centre.
- Number of samples taken from each participant.
- Scanners used to create digital whole slide images.
- Data augmentation techniques and/or generative adversarial networks used.
- For open-source datasets, the subset of samples used for validation and the year that samples were taken from participants.

**Diagnostic performance and metrics**

*Design recommendations:*

- The threshold at which outcomes are reported should be decided with consideration of the clinical task performed by the model. Consider whether sensitivity or specificity should be prioritised for this task.

*Reporting recommendations:*

- As a minimum, a confusion matrix should be provided, including the number of true positives, true negatives, false positives and false negatives.
- Metrics with clinical utility including sensitivity, specificity, AUROC, negative predictive value and positive predictive value.
- The threshold at which outcomes are reported.
- Model performance at whole-slide image-level.
- Outcomes according to prespecified subgroups (e.g. age, sex, ethnicity, cancer stage, cancer subtype).
- Clearly defined measures of variability (e.g. confidence intervals).

## Methods

### Search strategy and selection criteria

This systematic scoping review followed the Preferred Items for Systematic Reviews and Meta-Analysis guidelines extension for Scoping Reviews^44^. The protocol was published on Open Science Framework (https://osf.io/yacju) prior to conducting the review.

We screened 50 titles and abstracts for piloting purposes and to inform our search strategy. We subsequently searched for primary research articles published between 1^st^ January 2010 to 31^st^ October 2024, with no language restrictions. We chose 2010 as the starting date due to advancements in deep learning methods and increased availability of big data^48^. Using a combination of keywords related to ‘lung cancer’, ‘AI’, ‘validation’, ‘diagnosis’ and ‘pathology’, we systematically searched MEDLINE, Embase, Web of Science, IEEE, Engineering Village, and Association for Computing Machinery. The full search strategy for MEDLINE can be found in Supplementary material 1. Additionally, we searched ClinicalTrials.gov for study protocols, and BioRxiv and MedRxiv for preprint articles. We extended our systematic search to IBM and the first 200 studies in Google Scholar, ranked by relevance for studies that met our inclusion criteria. Snowballing was used to identify studies that may have been missed during the search process.

Studies were considered for inclusion if they provided evidence on the accuracy and utility of machine learning models for analysing histopathology or cytology images to aid in early lung cancer diagnosis. We did not impose restrictions in relation to study type as we anticipated that models would be at various stages of development and study type was one of our outcomes. Studies were excluded if they described the development of AI algorithms without any validation of their effectiveness; evaluated models unrelated to histopathology or cytology, were not designed for lung cancer diagnosis; were not machine learning models; only validated models internally; were review articles, or conference abstracts with insufficient information (e.g. lack of clarity on whether validation was internal or external).

### Data extraction, analysis and synthesis

After removing duplicates, two authors (SA and MK or MG) independently performed title and abstract screening as well as full-text screening to identify studies that met the inclusion criteria. Any conflicts were resolved independently by a third reviewer (JO). All stages of screening were conducted using Covidence systematic review software, Veritas Health Innovation, Melbourne, Australia. Data extraction was performed independently by at least two reviewers (SA and MK or MG) using a pre-designed data extraction template, which was included in the published protocol.

The extracted data included details related to the type of study used to validate models, AI task, model performance where available, evaluation metrics used, validation dataset size, number of datasets, technical diversity within datasets and dataset source (public or restricted). Public datasets are openly accessible to the public via online repositories, whereas restricted datasets are not openly accessible to the public and may require authorised access^49^. We additionally aimed to collect information on intended clinical setting, validation setting, and reporting. All authors of the included studies were contacted for missing information. Due to considerable heterogeneity in AI task, evaluation metrics used, unit of analysis and reporting, we used a narrative synthesis approach rather than a meta-analysis.

### Quality assessment

We conducted a quality assessment to further investigate the methodological robustness of validation studies. We modified the Quality Assessment of Diagnostic Accuracy Studies 2 (QUADAS-2) tool to better reflect specifics of AI and digital pathology^50^. Signalling questions were adapted with input from experts in AI and digital pathology (QUADAS-AI-P, see supplementary material 2). All five domains of QUADAS-AI-P are concerned with the external validation phase. Two authors (SA and MK or MG) independently conducted a quality assessment using the QUADAS-AI-P tool.

## Supplementary information

Supplementary Information

## Data Availability

The data that support the findings of this study are available from corresponding publications and are available from authors upon reasonable request.
