# Supplementary material for: Systematic scoping review of external validation studies of AI pathology models for lung cancer diagnosis
Source: NPJ Precis Oncol. 2025 Jun 7;9:166. doi: 10.1038/s41698-025-00940-7 (PMC12145442; doi:10.1038/s41698-025-00940-7)
Supplement: Supplementary file 1 — Supplementary Information [file 41698_2025_940_MOESM1_ESM.pdf]

## Supplementary Information

### Table of Contents

Supplementary Material 1: Example of a search strategy for bibliographic databases ........................ 2

Supplementary Material 2: Quality assessment tool of diagnostic accuracy studies tailored to artificial intelligence and digital pathology (QUADAS-AI-P) ................................................................ 5

Supplementary Material 3: Table showing the risk of bias and concerns regarding applicability for each of the included studies ........................................................................................................ 6

Supplementary Material 4: Table of methodological concerns .......................................................... 8

Supplementary Material 5: Funding sources of individual studies..................................................... 10

Supplementary Material 6: Preferred Reporting Items for Systematic reviews and Meta-Analyses extension for Scoping Reviews (PRISMA-ScR) Checklist .................................................................... 12

References ........................................................................................................................................ 14

**Supplementary Material 1: Example of a search strategy for bibliographic databases**

---

**Medline Search Strategy**

Ovid MEDLINE(R) ALL

<1946 to October 31, 2024>

| | | |
|---|---|---|
| 1 | validation.ab,kw,ti. | 304518 |
| 2 | "validat*".ab,kw,ti. | 747042 |
| 3 | accuracy.ab,kw,ti. | 554360 |
| 4 | "accura*".ab,kw,ti. | 1065166 |
| 5 | effectiveness.ab,kw,ti. | 591622 |
| 6 | "effective*".ab,kw,ti. | 2551876 |
| 7 | evaluation.ab,kw,ti. | 1457715 |
| 8 | "evaluat*".ab,kw,ti. | 4437488 |
| 9 | Validation Study/ | 109212 |
| 10 | "Sensitivity and Specificity"/ | 369325 |
| 11 | Evaluation Study/ | 261997 |
| 12 | 1 or 2 or 3 or 4 or 5 or 6 or 7 or 8 or 9 or 10 or 11 | 7680280 |
| 13 | artificial intelligence.ab,kw,ti. | 38218 |
| 14 | machine learning.ab,kw,ti. | 93351 |
| 15 | deep learning.ab,kw,ti. | 51275 |
| 16 | AI.ab,kw,ti. | 46131 |
| 17 | algorithm.ab,kw,ti. | 260562 |
| 18 | neural networks.ab,kw,ti. | 45572 |
| 19 | supervised learning.ab,kw,ti. | 5238 |
| 20 | Artificial Intelligence/ | 39848 |
| 21 | Diagnosis, Computer-Assisted/ or Machine Learning/ | 57596 |

| | | |
|---|---|---|
| 22 | Deep Learning/ | 16391 |
| 23 | Algorithms/ | 304764 |
| 24 | Neural Networks, Computer/ | 48535 |
| 25 | Pattern Recognition, Automated/ or Supervised Machine Learning/ or Image Processing, Computer-Assisted/ | 165673 |
| 26 | 13 or 14 or 15 or 16 or 17 or 18 or 19 or 20 or 21 or 22 or 23 or 24 or 25 | 770896 |
| 27 | pathology.ab,kw,ti. | 375577 |
| 28 | histopathology.ab,kw,ti. | 82088 |
| 29 | Pathology, Clinical/ or Pathology/ or Pathology, Surgical/ or Pathology, Molecular/ | 43400 |
| 30 | 27 or 28 or 29 | 461460 |
| 31 | Diagnosis.ab,kw,ti. | 1867730 |
| 32 | detection.ab,kw,ti. | 1093766 |
| 33 | computer-aided detection.ab,kw,ti. | 1920 |
| 34 | computer aided detection.ab,kw,ti. | 1920 |
| 35 | CAD.ab,kw,ti. | 50273 |
| 36 | Early Diagnosis/ or Diagnosis/ or Diagnosis, Computer-Assisted/ | 71471 |
| 37 | "diagnos*".ab,kw,ti. | 3058578 |
| 38 | "detect*".ab,kw,ti. | 2809926 |
| 39 | 31 or 32 or 33 or 34 or 35 or 36 or 37 or 38 | 5422041 |
| 40 | lung cancer.ab,kw,ti. | 201460 |
| 41 | non-small cell lung cancer.ab,kw,ti. | 77187 |
| 42 | non small cell lung cancer.ab,kw,ti. | 77187 |
| 43 | NSCLC.ab,kw,ti. | 60568 |
| 44 | SCLC.ab,kw,ti. | 10034 |
| 45 | small cell lung cancer.ab,kw,ti. | 87622 |
| 46 | squamous cell carcinoma.ab,kw,ti. | 110667 |

| | | |
|---|---|---|
| 47 | large cell carcinoma.ab,kw,ti. | 1795 |
| 48 | adenocarcinoma.ab,kw,ti. | 163109 |
| 49 | lung carcinoma.ab,kw,ti. | 20568 |
| 50 | lung neoplasms.ab,kw,ti. | 5819 |
| 51 | epidermoid carcinoma.ab,kw,ti. | 3860 |
| 52 | pulmonary cancer.ab,kw,ti. | 1145 |
| 53 | pulmonary neoplasms.ab,kw,ti | 566 |
| 54 | pulmonary carcinoma.ab,kw,ti. | 1181 |
| 55 | oat cell lung cancer.ab,kw,ti. | 50 |
| 56 | lung tumour.ab,kw,ti. | 1062 |
| 57 | Lung Neoplasms/ | 259177 |
| 58 | Carcinoma, Non-Small-Cell Lung/ | 71086 |
| 59 | Carcinoma, Small Cell/ | 17563 |
| 60 | Carcinoma, Squamous Cell/ | 141251 |
| 61 | Carcinoma, Large Cell/ | 2576 |
| 62 | "Adenocarcinoma of Lung"/ | 10726 |
| 63 | 40 or 41 or 42 or 43 or 44 or 45 or 46 or 47 or 48 or 49 or 50 or 51 or 52 or 53 or 54 or 55 or 56 or 57 or 58 or 59 or 60 or 61 or 62 | 618283 |
| 64 | 12 and 26 and 30 and 39 and 63 | 410 |
| 65 | 12 and 26 and 30 and 39 and 63 | 410 |
| 66 | limit 64 to yr="2010-2024" | 351 |

---

The most recent search was executed on January 12th 2025.

**Supplementary Material 2: Quality assessment tool of diagnostic accuracy studies tailored to artificial intelligence and digital pathology (QUADAS-AI-P)**

**Participant selection/study design**

- Did the validation dataset include more than 100 samples?
- If not, did the validation dataset consist of more than 50 unique samples?
- Was the biopsy case selection representative of the condition being assessed in the study?
- Was a consecutive or random sample of participants enrolled?
- Was a case-control design or enriched cohort study avoided?
- Was the sample diverse in terms of sex, age, sociodemographic status and ethnicity?
- Was the study cohort generalizable to the target population?
- Was the algorithm validated in the setting (country, healthcare setting) it is intended to be deployed in?

**Image selection**

- Did the validation dataset include the complete digitised biopsy section?
- Did the validation dataset consist of images taken from multiple centres?
- Did the validation dataset consist of images prepared using different scanners and were the number of images produced by each scanner roughly equal?
- If different scanners were not used and algorithms were validated using single-centre data, were data augmentation techniques used?
- Was an open-source dataset used?
- Was validation performed using scanners that were not used when training the model?

**Reference standard**

- Was the gold-standard (histopathological assessment by a pathologist) used as the reference standard?
- Was there more than one pathologist involved in making a diagnosis?
- Were pathologists blinded to the results of the algorithm when interpreting biopsy samples?
- Was assessment by pathologists based on digital biopsy slides?

**Index test**

- Were algorithm results interpreted without knowledge of the result of the reference standard?

**Flow and timing**

- Was there an appropriate time interval between application of the algorithm and the reference standard?
- Were all participants included in the analysis?
- Did all participants receive a reference standard?
- Did all participants receive the same reference standard?

**Supplementary Material 3: Table showing the risk of bias and concerns regarding applicability for each of the included studies**

| Study Reference | Risk of bias | | | | | Concerns regarding applicability | | |
|---|---|---|---|---|---|---|---|---|
| | Participant selection/ study design | Image selection | Reference standard | Index test | Flow and timing | Participant selection | Index test | Target condition |
| Bilaloglu *et al*. 2019[1] | High | High | Unclear | Low | Unclear | Unclear | Low | Unclear |
| Borras Ferris *et al*. 2024[2] | High | Unclear | Unclear | Unclear | Unclear | Low | Low | Low |
| Cao *et al*. 2023[3] | High | Low | High | Unclear | Unclear | Unclear | Low | Low |
| Chen *et al*. 2022[4] | Unclear | High | Unclear | Low | Low | Unclear | Low | Low |
| Coudray *et al*. 2018[5] | High | High | Unclear | Unclear | Unclear | Unclear | Low | Low |
| Gertych *et al*. 2019[6] | High | High | Unclear | Unclear | Low | Unclear | Low | Low |
| Hari *et al*. 2021[7] | High | High | Unclear | Low | Unclear | Unclear | Low | Unclear |
| Kanavati *et al*. 2020[8] | Unclear | Unclear | Unclear | Unclear | Unclear | Unclear | Low | Low |
| Kanavati *et al*. 2021[9] | High | Unclear | Low | Unclear | Low | Unclear | Low | Low |
| Le Page *et al*. 2021[10] | High | High | High | Unclear | Unclear | Unclear | Low | Low |
| Lu *et al*. 2021[11] | High | Low | High | Unclear | Unclear | Unclear | Low | Unclear |
| Mukashyaka *et al*. 2024[12] | High | High | Unclear | Unclear | Unclear | Unclear | Low | Low |
| Noorbakhsh *et al*. 2020[13] | High | High | Unclear | Low | Unclear | Unclear | Low | Low |
| Quiros *et al*. 2024[14] | High | High | Unclear | Unclear | Unclear | Unclear | Low | Unclear |
| Sakamoto *et al*. 2022[15] | High | Low | Low | Low | Low | Unclear | Low | Low |
| Sharma *et al*. 2024[16] | High | High | Unclear | Unclear | Unclear | Unclear | Low | Low |
| Swiderska-Chadaj *et al*. 2020[17] | High | Low | Low | Unclear | Unclear | Unclear | Low | Low |
| Vorontsov *et al*. 2024[18] | High | Unclear | Unclear | Unclear | Unclear | Unclear | Unclear | Unclear |

| | | | | | | | | |
|---|---|---|---|---|---|---|---|---|
| Wang *et al*. 2019[19] | High | Unclear | Low | Unclear | Low | Low | Low | Low |
| Wang et al. 2023[20] | High | Unclear | Unclear | Unclear | Unclear | Unclear | Low | Low |
| Yang et al. 2021[21] | Unclear | Unclear | Low | Low | Low | Low | Low | Low |
| Yu et al. 2020[22] | High | High | Unclear | Low | Low | Low | Low | High |

**Supplementary Material 4: Table of methodological concerns**

| Key methodological concerns |
|---|
| **Algorithm** |
| • Intended clinical setting was not clearly defined for the majority of studies<br>• Lack of clarity on how models will fit into existing pathways (as an aid, triage, or replacement for the clinician) |
| **Study design** |
| • High proportion of studies used a retrospective case-control design<br>• Majority of studies used small datasets consisting of fewer than 500 samples<br>• A large number of studies used data from a single centre only<br>Poor reporting of:<br>• The number of datasets that samples were taken from<br>• Validation setting<br>• Study type |
| **Population/participant selection** |
| • Target population often not defined<br>• Non-diverse and unrepresentative datasets used<br>• The number of participants from which samples were taken from was often unclear<br>Poor reporting of:<br>• Participant characteristics<br>• Participant enrolment |
| **Image selection** |
| • A large proportion of studies did not account for technical variation that may occur across different centres<br>Poor reporting of:<br>• Whether any technical diversity was achieved<br>• Reference standard<br>• Scanners used for training and validation |
| **Diagnostic performance and metrics** |

- High heterogeneity in metrics used, limiting ability to compare models

Poor reporting of:

- Clinically meaningful measures such as sensitivity and specificity
- Measures of variability (e.g. confidence intervals)

**Supplementary Material 5: Funding sources of individual studies**

| Study Reference | Funding source(s) |
|---|---|
| Bilaloglu *et al*. 2019[1] | • Cancer Center Support Grant, NYU School of Medicine Laura and Isaac Perlmutter Cancer Center |
| Borras Ferris *et al*. 2024[2] | • European Union's Horizon 2020 research and innovation program |
| Cao *et al*. 2023[3] | • National Natural Science Foundation of China<br>• Fundamental Research Funds for the Central Universities, China |
| Chen *et al*. 2022[4] | • National Key R&D Program of China<br>• National Natural Science Foundation of China<br>• Guangdong Natural Science Foundation |
| Coudray *et al*. 2018[5] | • Cancer Center Support Grant, NYU School of Medicine Laura and Isaac Perlmutter Cancer Center. |
| Gertych *et al*. 2019[6] | • Precision Health Grant at Cedars-Sinai Medical Center<br>• Department of Surgery at Cedars-Sinai Medical Center<br>• CTSI grant, Cedars-Sinai<br>• National Science Centre, Poland |
| Hari *et al*. 2021[7] | • R37CA222574<br>• R01CA2227388<br>• P50CA101942<br>• Dunkin' Donuts Breakthrough Grant |
| Kanavati *et al*. 2020[8] | - |
| Kanavati *et al*. 2021[9] | - |
| Le Page *et al*. 2021[10] | - |
| Lu *et al*. 2021[11] | • BWH Pathology<br>• Google Cloud Research Grant<br>• Nvidia GPU Grant Program<br>• NSF Graduate Fellowship<br>• National Institutes of Health |
| Mukashyaka *et al*. 2024[12] | • Jackson laboratory<br>• National Institutes of Health/National Cancer Institute |
| Noorbakhsh *et al*. 2020[13] | • National Institutes of Health Cloud Credits Model Pilot, Google Cloud<br>• National Cancer Institute |
| Quiros *et al*. 2024[14] | • Cancer Center Support Grant, NYU School of Medicine Laura and Isaac Perlmutter Cancer Center<br>• EP/R018634/1<br>• BB/V016067/1<br>• European Union's Horizon 2020 research and innovation programme<br>• Mazumdar-Shaw Molecular Pathology Chair endowment, University of Glasgow<br>• School of Computing Science, University of Glasgow<br>• Openshift GPU cluster management team |
| Sakamoto *et al*. 2022[15] | • New Energy and Industrial Technology Development Organisation |

| | |
|---|---|
| Sharma *et al*. 2024[16] | - |
| Swiderska-Chadaj *et al*. 2020[17] | • Precision Health Grant at Cedars-Sinai Medical Center<br>• Department of Surgery at Cedars-Sinai Medical Center<br>• National Science Centre, Poland |
| Vorontsov *et al*. 2024[18] | • Cancer Center Support Grant, National Institutes of Health/National Cancer Institute |
| Wang *et al*. 2019[19] | • National Institutes of Health<br>• Cancer Prevention and Research Institute of Texas |
| Wang et al. 2023[20] | • National Institutes of Health<br>• Cancer Prevention and Research Institute of Texas |
| Yang et al. 2021[21] | • National Key R&D Program of China<br>• National Natural Science Foundation of China<br>• Guangdong Natural Science Foundation<br>• Support Scheme of Guangzhou for Leading Talents in Innovation and Entrepreneurship |
| Yu et al. 2020[22] | • National Cancer Institute, National Institutes of Health<br>• National Human Genome Research Institute, National Institutes of Health<br>• Mobilize Center, Stanford University<br>• Harvard Data Science Fellowship<br>• Harvard Medical School Center for Computational Biomedicine Award |

**Supplementary Material 6: Preferred Reporting Items for Systematic reviews and Meta-Analyses extension for Scoping Reviews (PRISMA-ScR) Checklist**

| SECTION | ITEM | PRISMA-ScR CHECKLIST ITEM | REPORTED ON PAGE # |
|---|---|---|---|
| **TITLE** | | | |
| Title | 1 | Identify the report as a scoping review. | 1 |
| **ABSTRACT** | | | |
| Structured summary | 2 | Provide a structured summary that includes (as applicable): background, objectives, eligibility criteria, sources of evidence, charting methods, results, and conclusions that relate to the review questions and objectives. | 1 |
| **INTRODUCTION** | | | |
| Rationale | 3 | Describe the rationale for the review in the context of what is already known. Explain why the review questions/objectives lend themselves to a scoping review approach. | 2 |
| Objectives | 4 | Provide an explicit statement of the questions and objectives being addressed with reference to their key elements (e.g., population or participants, concepts, and context) or other relevant key elements used to conceptualize the review questions and/or objectives. | 2, protocol (5, 6) |
| **METHODS** | | | |
| Protocol and registration | 5 | Indicate whether a review protocol exists; state if and where it can be accessed (e.g., a Web address); and if available, provide registration information, including the registration number. | 8 |
| Eligibility criteria | 6 | Specify characteristics of the sources of evidence used as eligibility criteria (e.g., years considered, language, and publication status), and provide a rationale. | 8 |
| Information sources* | 7 | Describe all information sources in the search (e.g., databases with dates of coverage and contact with authors to identify additional sources), as well as the date the most recent search was executed. | 8, 10, Supplementary material 1 |
| Search | 8 | Present the full electronic search strategy for at least 1 database, including any limits used, such that it could be repeated. | Supplementary material 1, 8 |
| Selection of sources of evidence† | 9 | State the process for selecting sources of evidence (i.e., screening and eligibility) included in the scoping review. | 8 |
| Data charting process‡ | 10 | Describe the methods of charting data from the included sources of evidence (e.g., calibrated forms or forms that have been tested by the team before their use, and whether data charting was done independently or in duplicate) and any processes for obtaining and confirming data from investigators. | 8, 10 |

| SECTION | ITEM | PRISMA-ScR CHECKLIST ITEM | REPORTED ON PAGE # |
|---|---|---|---|
| Data items | 11 | List and define all variables for which data were sought and any assumptions and simplifications made. | 10, Supplementary material 2, protocol (8, 9) |
| Critical appraisal of individual sources of evidence§ | 12 | If done, provide a rationale for conducting a critical appraisal of included sources of evidence; describe the methods used and how this information was used in any data synthesis (if appropriate). | 10 |
| Synthesis of results | 13 | Describe the methods of handling and summarizing the data that were charted. | 8, 10 |
| **RESULTS** | | | |
| Selection of sources of evidence | 14 | Give numbers of sources of evidence screened, assessed for eligibility, and included in the review, with reasons for exclusions at each stage, ideally using a flow diagram. | Figure 1, 2 |
| Characteristics of sources of evidence | 15 | For each source of evidence, present characteristics for which data were charted and provide the citations. | Table 1 |
| Critical appraisal within sources of evidence | 16 | If done, present data on critical appraisal of included sources of evidence (see item 12). | Figure 3, Supplementary material 3 |
| Results of individual sources of evidence | 17 | For each included source of evidence, present the relevant data that were charted that relate to the review questions and objectives. | Table 1, table 2, 3, 4, 5, 6 |
| Synthesis of results | 18 | Summarize and/or present the charting results as they relate to the review questions and objectives. | 2, 3, 4, 5, 6 |
| **DISCUSSION** | | | |
| Summary of evidence | 19 | Summarize the main results (including an overview of concepts, themes, and types of evidence available), link to the review questions and objectives, and consider the relevance to key groups. | 6, 7, 8 |
| Limitations | 20 | Discuss the limitations of the scoping review process. | 8 |
| Conclusions | 21 | Provide a general interpretation of the results with respect to the review questions and objectives, as well as potential implications and/or next steps. | 8 |
| **FUNDING** | | | |
| Funding | 22 | Describe sources of funding for the included sources of evidence, as well as sources of funding for the scoping review. Describe the role of the funders of the scoping review. | 11, Supplementary material 5 |

## References


1 Bilaloglu, S. *et al.* Efficient pan-cancer whole-slide image classification and outlier detection using convolutional neural networks. *bioRxiv*, 633123; 10.1101/633123 (2019).

2 Borras Ferris, L. *et al.* A full pipeline to analyze lung histopathology images. *Proceedings of SPIE - Progress in Biomedical Optics and Imaging*. **12933**; 10.1117/12.3006708 (2024).

3 Cao, L. *et al.* E2EFP-MIL: End-to-end and high-generalizability weakly supervised deep convolutional network for lung cancer classification from whole slide image. *Med Image Anal*. **88**, 102837; 10.1016/j.media.2023.102837 (2023).

4 Chen, Y. *et al.* A whole-slide image (WSI)-based immunohistochemical feature prediction system improves the subtyping of lung cancer. *Lung Cancer*. **165**, 18-27; 10.1016/j.lungcan.2022.01.005 (2022).

5 Coudray, N. *et al.* Classification and mutation prediction from non-small cell lung cancer histopathology images using deep learning. *Nat Med*. **24**, 1559-1567; 10.1038/s41591-018-0177-5 (2018).

6 Gertych, A. *et al.* Convolutional neural networks can accurately distinguish four histologic growth patterns of lung adenocarcinoma in digital slides. *Scientific Reports*. **9**, 1483; 10.1038/s41598-018-37638-9 (2019).

7 Hari, S. N. *et al.* Examining Batch Effect in Histopathology as a Distributionally Robust Optimization Problem. *bioRxiv*; 10.1101/2021.09.14.460365 (2021).

8 Kanavati, F. *et al.* Weakly-supervised learning for lung carcinoma classification using deep learning. *Scientific Reports*. **10**, 9297; 10.1038/s41598-020-66333-x (2020).

9 Kanavati, F. *et al.* A deep learning model for the classification of indeterminate lung carcinoma in biopsy whole slide images. *Scientific Reports*. **11**, 8110; 10.1038/s41598-021-87644-7 (2021).

10 Le Page, A. L. *et al.* Using a convolutional neural network for classification of squamous and non-squamous non-small cell lung cancer based on diagnostic histopathology HES images. *Sci Rep*. **11**, 23912; 10.1038/s41598-021-03206-x (2021).

11 Lu, M. Y. *et al.* Data-efficient and weakly supervised computational pathology on whole-slide images. *Nature Biomedical Engineering*. **5**, 555-570; 10.1038/s41551-020-00682-w (2021).

12 Mukashyaka, P., Sheridan, T. B., Foroughi pour, A. & Chuang, J. H. SAMPLER: unsupervised representations for rapid analysis of whole slide tissue images. *eBioMedicine*. **99**, 104908; 10.1016/j.ebiom.2023.104908 (2024).

13 Noorbakhsh, J. *et al.* Deep learning-based cross-classifications reveal conserved spatial behaviors within tumor histological images. *Nature Communications*. **11**, 6367; 10.1038/s41467-020-20030-5 (2020).

14 Claudio Quiros, A. *et al.* Mapping the landscape of histomorphological cancer phenotypes using self-supervised learning on unannotated pathology slides. *Nature Communications*. **15**, 4596; 10.1038/s41467-024-48666-7 (2024).

15 Sakamoto, T. *et al.* A collaborative workflow between pathologists and deep learning for the evaluation of tumour cellularity in lung adenocarcinoma. *Histopathology*. **81**, 758-769; 10.1111/his.14779 (2022).

16 Sharma, R., Kumar, S., Shrivastava, A. & Bhatt, T. Optimizing Knowledge Transfer in Sequential Models: Leveraging Residual Connections in Flow Transfer Learning for Lung Cancer Classification. *Proceedings of the Fourteenth Indian Conference on Computer Vision, Graphics and Image Processing*; 10.1145/3627631.3627663 (2024).

17 Swiderska-Chadaj, Z. *et al.* A deep learning approach to assess the predominant tumor growth pattern in whole-slide images of lung adenocarcinoma. *Medical Imaging 2020: Digital Pathology*; 10.1117/12.2549742 (2020).

18 Vorontsov, E. *et al.* A foundation model for clinical-grade computational pathology and rare cancers detection. *Nature Medicine*. **30**, 2924-2935; 10.1038/s41591-024-03141-0 (2024).

19 Wang, S. *et al.* ConvPath: A software tool for lung adenocarcinoma digital pathological image analysis aided by a convolutional neural network. *EBioMedicine*. **50**, 103-110; 10.1016/j.ebiom.2019.10.033 (2019).
20 Wang, S. *et al.* Deep learning of cell spatial organizations identifies clinically relevant insights in tissue images. *Nature Communications*. **14**, 7872; 10.1038/s41467-023-43172-8 (2023).
21 Yang, H. *et al.* Deep learning-based six-type classifier for lung cancer and mimics from histopathological whole slide images: a retrospective study. *BMC Med*. **19**, 80; 10.1186/s12916-021-01953-2 (2021).
22 Yu, K. H. *et al.* Classifying non-small cell lung cancer types and transcriptomic subtypes using convolutional neural networks. *J Am Med Inform Assoc*. **27**, 757-769; 10.1093/jamia/ocz230 (2020).
